# Clinical Validation and Standardization of the Brain Health Assessment in the Tablet‐Based Cognitive Assessment Tool (BHA‐TabCAT) in Peruvian Older Adults

**DOI:** 10.1002/alz70857_105247

**Published:** 2025-12-25

**Authors:** Andres Muñoz‐Najar, Manuel Montemurro, Maria del Carmen Tejada, Claudia Rivera‐Fernandez, Miguel Sanchez‐Fernandez, Nilton Custodio, Serggio Lanata, Marcio Soto‐Añari

**Affiliations:** ^1^ Universidad Catolica San Pablo, Arequipa, Peru; ^2^ Instituto de Bienestar socioemocional, Universidad del Desarrollo, Santiago, Santiago, Chile; ^3^ Universidad del Alba, Santiago, Chile; ^4^ nstituto de Bienestar Socioemocional ‐ Universidad del Desarrollo, Santiago, Santiago, Chile; ^5^ Universidad Adolfo Ibáñez, Santiago, Chile; ^6^ Universidad Tecnológica del Perú, Arequipa, Peru; ^7^ Global Brain Health Institute, Arequipa, Peru; ^8^ Unidad de Investigación y Docencia, Equilibria, Lima, Lima, Peru; ^9^ Unidad de diagnóstico de deterioro cognitivo y prevención de demencia, Instituto Peruano de Neurociencias, Lima, Perú, Lima, Lima, Peru; ^10^ Memory and Aging Center, UCSF Weill Institute for Neurosciences, University of California, San Francisco, San Francisco, CA, USA; ^11^ Global Brain Health Institute, San Francisco, CA, USA; ^12^ Universidad Católica San Pablo, Arequipa, Peru

## Abstract

**Background:**

Detecting cognitive decline presents a significant challenge for researchers and clinicians, particularly in older adults with low educational levels or different sociocultural characteristics. Additionally, traditional normative data may under‐ or overestimate preliminary diagnoses in these populations, due its non‐use of measures which impact cognitive performance as years of schooling or age. This study aimed to validate and standardize the Brain Health Assessment Tablet‐Based Cognitive Assessment Tool (BHA‐TabCAT) among Andean Peruvian older adults.

**Method:**

A total of 258 participants between the ages of 54 and 91 were assessed with BHA‐TabCAT, the Mini Mental State Examination (MMSE) and the Rowland Universal (RUDAS). Participants were evaluated in the rural district of Pampacolca, and in the urban district of Arequipa, both located in the Andean city of Arequipa ‐ Peru. All participants were assigned a clinical diagnosis group (cognitively health or mild cognitive impairment). We used regression‐based norming procedures, including sex, place of residence, age and years of schooling as covariates. We followed the recommendations of Tsoy et al. (2021).

**Result:**

Initially, the BHA‐CS's analysis was performed considering weighted scores of significant and non‐significant sociodemographic predictors. AUC captures the 75.3% of positive cases, against the MMSE (Specificity = 81.7%, Sensitivity = 48.6%, Accuracy = 72.5%, AUC = 66.4%) and RUDAS (Specificity = 76.3%, Sensitivity = 62.5%, Accuracy = 72.5% AUC = 71.4%). A second analysis was conducted using only the weighted scores of significant sociodemographic predictors. AUC of the BHA‐CS captures 77.4% of the positive cases against the MMSE (Specificity = 77.4%, Sensitivity = 52.8%, Accuracy = 70.5%, AUC = 66.6%) and RUDAS (Specificity = 75.3%, Sensitivity = 66.7% Accuracy = 72.9%, AUC = 71.9%).

**Conclusion:**

The BHA‐TabCAT shows better performance than the MMSE and RUDAS in detecting cognitive decline, especially when its scoring and interpretation consider relevant covariates such as years of education, age, place of residence, and gender. This provides improved opportunities to accurately identify the preclinical and prodromal stages of cognitive decline in both urban and rural contexts in Peru.

